# Supplementary material for: Response nonlinearities in networks of spiking neurons
Source: PLoS Comput Biol. 2020 Sep 17;16(9):e1008165. doi: 10.1371/journal.pcbi.1008165 (PMC7524009; doi:10.1371/journal.pcbi.1008165)
Supplement: S1 Text — (PDF) [file pcbi.1008165.s001.pdf]

Response nonlinearities in networks of spiking neurons

Alessandro Sanzeni[1,2], Mark H. Histed[1], Nicolas Brunel[2,3,*]

1 National institute of Mental Health Intramural Program, NIH, Bethesda, MD, USA
2 Department of Neurobiology, Duke University, Durham, NC, USA
3 Department of Physics, Duke University, Durham, NC, USA

* nicolas.brunel@duke.edu

Supporting information

S1 Text: Admissibility conditions for model B solutions at finite coupling

In the main text, we have shown that there are 5 types of solutions which are admissible in the strong coupling limit of model B. At finite coupling, only solutions producing rates below saturation are admissible. Using this constraint, for every solution described in Eqs (24), (26) and (27) of the main text, we derive the following admissibility conditions.

Solutions of s1 type appear if connectivity is such that

$$\frac{\alpha_I}{\alpha_E} < \frac{g_I}{g_E} < 1 \text{ or if } 1 < \frac{g_I}{g_E} < \frac{\alpha_I}{\alpha_E}\,. \tag{1}$$

Moreover, the external input must satisfy the condition

$$\nu_X \tau_{rp} < \min\left\{\frac{g_E - g_I}{g_I\alpha_E - g_E\alpha_I}, \frac{\gamma(g_E - g_I)}{\alpha_E - \alpha_I}\right\}. \tag{2}$$

i.e. none of the two populations is saturated.

Solutions of s2 type appear only if

$$\frac{g_I}{g_E} < \frac{\alpha_I}{\alpha_E} \text{ and } \nu_X \tau_{rp} < \frac{\gamma g_I}{\alpha_I}\,. \tag{3}$$

Solutions of s3 type appear only if

$$\max\left\{\frac{\gamma g_E - 1}{\alpha_E}, \frac{\gamma g_I - 1}{\alpha_I}\right\} < \nu_X \tau_{rp} \tag{4}$$

Solutions of s4 type appear only if

$$\frac{\gamma g_E - 1}{\alpha_E} < \nu_X \tau_{rp} < \min\left\{\frac{\gamma g_E}{\alpha_E}, \frac{\gamma(g_E - g_I)}{\alpha_E - \alpha_I}\right\} \text{ and } \frac{\alpha_I}{\alpha_E} < 1 \tag{5}$$

or if

$$\max\left\{\frac{\gamma(g_E - g_I)}{\alpha_E - \alpha_I}, \frac{\gamma g_E - 1}{\alpha_E}\right\} < \nu_X \tau_{rp} < \frac{\gamma g_E}{\alpha_E} \text{ and } 1 < \frac{\alpha_I}{\alpha_E} \tag{6}$$

Solutions of s5 type appear only if

$$\frac{g_E - g_I}{g_I\alpha_E - g_E\alpha_I} < \nu_X \tau_{rp} < \frac{\gamma g_I - 1}{\alpha_I} \text{ and } \frac{\alpha_I}{\alpha_E} < \frac{g_I}{g_E} \tag{7}$$

or if

$$\nu_X \tau_{rp} < \min\left\{\frac{\gamma g_I - 1}{\alpha_I}, \frac{g_E - g_I}{g_I \alpha_E - g_E \alpha_I}\right\} \text{ and } \frac{g_I}{g_E} < \frac{\alpha_I}{\alpha_E} \tag{8}$$

Combining the above inequalities we find the following relations between the different solutions

- Conditions on s3 and s5 solutions are mutually exclusive, which implies that only one of the two solution types can appear at any given value of ν_X.
- When solutions s1 and s2 coexist, s4 solutions are excluded.

Combining these two results, we find that for any value of ν_X there can be at most three solutions.
