# Supplementary material for: Response nonlinearities in networks of spiking neurons
Source: PLoS Comput Biol. 2020 Sep 17;16(9):e1008165. doi: 10.1371/journal.pcbi.1008165 (PMC7524009; doi:10.1371/journal.pcbi.1008165)
Supplement: S2 Text — (PDF) [file pcbi.1008165.s002.pdf]

Response nonlinearities in networks of spiking neurons

Alessandro Sanzeni[1,2], Mark H. Histed[1], Nicolas Brunel[2,3,*]

1 National institute of Mental Health Intramural Program, NIH, Bethesda, MD, USA
2 Department of Neurobiology, Duke University, Durham, NC, USA
3 Department of Physics, Duke University, Durham, NC, USA

* nicolas.brunel@duke.edu

Supporting information

S2 Text: Effects of shot noise on network response

In the main text, we have classified response nonlinearities in networks of spiking neurons using mathematical relations (specifically, Eq. (3) of the main text) which have been derived using the diffusion approximation of synaptic inputs. In this section, we show that our results remain valid when the shot-noise structure of synaptic inputs is taken into account.

The response dynamics of a LIF neuron driven by a shot-noise process has been investigated by several authors [1, 2]. The model used in [2] is analogous to the one we described in the method section of the main text, but features exponentially distributed J_{ij} (Eq. (2) of the main text). This modification, which is biologically plausible since post-synaptic potential amplitudes in cortex are broadly distributed with CVs close to 1 (see e.g. Table 1 in [3]), allows to solve analytically the master equation of the probability distribution of the membrane potential, without resorting to the diffusion approximation. Richardson and Swarbrick [2] have shown that the response of a single neuron driven by a shot-noise process is given by

$$\begin{aligned} \nu &= \left[\tau_{rp} + \tau \int_0^{\sigma/J_E} \frac{dx}{x} e^{W(x)} \left(\frac{e^{\frac{\theta}{\sigma}x}}{1 - \frac{J_E}{\sigma}x} - e^{\frac{V_r}{\sigma}x} \right) \right]^{-1} , \\ W(x) &= \tau R_E \log(1 - \frac{J_E}{\sigma}x) + \tau R_I \log(1 + \frac{J_I}{\sigma}x) , \end{aligned} \tag{1}$$

where J_E and J_I are the mean excitatory and inhibitory synaptic efficacy, R_E and R_I are the total firing rate produced by the presynaptic excitatory and inhibitory neurons, whilst

$$\sigma^2 = 2(J_E^2 R_E + J_I^2 R_I) . \tag{2}$$

In what follows we use Eq. (1) to study how shot-noise affects the network response. We perform our analysis in the framework of model A, where

$$R_E = K(\nu_X + \nu) , \quad R_I = \gamma K \nu , \quad J_E = J , \quad J_I = gJ . \tag{3}$$

Analogously to what happens with the diffusion approximation, the network response is obtained solving self-consistently Eq. (1); example solutions are shown in Fig. A. Over a wide range of parameters, these solutions follow those predicted using the diffusion approximation, i.e. solving Eq. (3) of the main text, with quantitative discrepancies limited to a few spk/s.

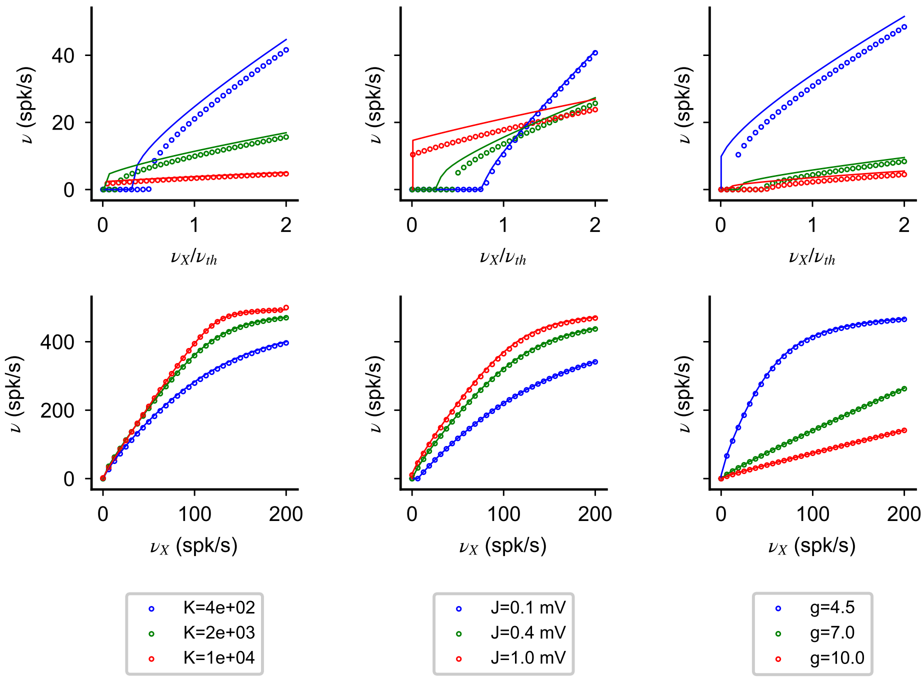

Fig A. Effects of shot noise on network response. Response of networks of spiking neurons computed using the mean field formalism with a full description of the shot-noise statistics of synaptic input (Eq. (1), circles) as a function of input strength for various network parameters. Over the explored range of parameters, responses are similar to those obtained using the diffusion approximation of synaptic input (continuous lines, Eq. (3) of the main text but, as discussed in the text, with σ given by Eq. (2). Discrepancies between the two models are larger when the assumptions of the diffusion approximation are more strongly violated, i.e. at low K or large J and g, but are at most a few spk/s. In the figure, the first row shows a close up of the second in the region of response-onset. Unless otherwise specified, parameters are: $K = 10^3$, $J = 0.5$mV, and $g = 5$.

The close similarity of network responses computed with and without the diffusion approximation can be understood as follows. Expanding Eq. (1) and keeping all the terms of order up to $1/\sqrt{K}$, we get

$$\frac{1}{\nu} = \tau_{rp} + \tau\sqrt{\pi}\int_{u_{min}}^{u_{max}} e^{x^2}\left[1+\mathrm{erf}(x)\right]\,dx + \frac{\tau J}{\sigma}F(u_{max}) - \frac{\tau^2 R_E J_E^3 - \tau^2 R_I J_I^3}{\sigma^3}\left[G(u_{max}) - G(u_{min})\right] , \quad (4)$$

with

$$F(x) = \sqrt{\pi}e^{x^2}\left[\mathrm{erf}\,(x)+1\right] , \quad G(x) = \frac{2}{3}\left[(2x^2+1)F(x)+2x\right] . \quad (5)$$

The first two terms on the right-hand side of Eq. (4) represent $1/\nu$ computed, under the diffusion approximation, for a neuron driven by a synaptic input as the one in Eq. (2) of the main text but with exponentially distributed J_{ij}. These two terms are analogous to those in Eq. (3) of the main text, but with σ^2 given by Eq. (2), i.e. multiplied by a factor two with respect to the case of homogeneous J_{ij} (Eq. (5) of the main text). This difference comes from the fact that, for exponentially distributed J_{ij} and under the diffusion approximation, the noise component of the synaptic inputs is proportional to $\langle J_{ij}^2\rangle = 2J^2$.

Eq. (4), with respect to Eq. (3) of the main text, has two additional terms (proportional to J/σ and RJ^3/σ^3, respectively) which represent corrections produced by the shot-noise statistics of the input. We now analyze the effect of these terms on response nonlinearities at finite K following the approach of the main text, i.e. investigating separately the different regimes of activity.

In the high input/high rate regime, $u_{max} \ll -1$, $F(x) \sim 1/x$ and Eq. (4) reduces to

$$\begin{aligned}(1-\tau_{rp}\nu)\left[\nu_X - \nu_{th} - (g\gamma-1)\nu\right] = \epsilon\nu(1-\Delta_{high}(\nu)) , \quad \epsilon = \frac{\theta - V_r}{KJ} , \\ \Delta_{high}(\nu) = \frac{J}{\theta - V_r} + \frac{1}{3}\frac{(\nu_X - (g^3\gamma-1)\nu)\left[\nu_X - \nu_{th} - (g\gamma-1)\nu\right]}{\left[\nu_X + (g^2\gamma+1)\nu\right]^2} ,\end{aligned} \quad (6)$$

which, when $\Delta_{high}(\nu)$ is negligible, is analogous to the equation obtained using the diffusion approximation (Eq. (13) of the main text). To classify response nonlinearities, we solve Eq. (6) with a perturbative expansion $\nu = \nu_0 + \epsilon\nu + \ldots$. As with the diffusion approximation, there are two solutions at order $\epsilon = 0$ (strong coupling limit) in Eq. (6): the balanced-state solution $\nu_0 = (\nu_X - \nu_{th})/(\gamma g - 1)$ and the saturated solution $\nu_0 = 1/\tau_{rp}$. Using the balanced-state solution in the ϵ expansion, we get

$$\nu_0 = \frac{\nu_X - \nu_{th}}{\gamma g - 1} , \quad \nu_1 = -\frac{\nu_0}{(\gamma g - 1)\,(1-\tau_{rp}\nu_0)} , \quad (7)$$

which is exactly what we found with the diffusion approximation (Eq. (20) of the main text). Deviations produced by the non-Gaussian input (terms proportional to Δ_{high}) appear at the second order in ϵ and hence do not affect the classification of nonlinearities (supralinear vs sublinear solutions) done in the main text. To understand if the non-Gaussian input influence the number of solutions at a given value of ν_X, we computed first order correction to the saturated solution and found

$$\nu_0 = \frac{1}{\tau_r p} , \quad \nu_1 = -\frac{\nu_0\,(1-\Delta_{high}(\nu_0))}{\tau_{rp}\,(\nu_X - \nu_{th}) - (\gamma g - 1)} . \quad (8)$$

Therefore, unlike what happens for balanced-state solution, corrections due to shot-noise appear at the first order in the ϵ expansion. However, these corrections do

not modify the sign of ν_1 and hence, as discussed in the main text, the number of admissible solutions in the network response. In fact, in $\Delta_{high}(\nu_0)$, the first term is small (typical values are $J \in [0.1-1]$mV, $\theta - V_r \sim$10mV) and the maximum value of the second term in $\Delta_{high}(\nu_0)$ is 1/3.

In the low input/low rate regime, $u_{max} \gg 1$, $F(x) \sim 2\sqrt{\pi}\exp(x^2)$ and Eq. (4) reduces to

$$\begin{aligned} \tau\nu &= \frac{u_{max}}{\sqrt{\pi}} e^{-u_{max}^2} \frac{1}{1+\Delta_{low}(\nu)}\,, \\ \Delta_{low}(\nu) &= \frac{u_{max}}{\sqrt{K}} \sqrt{\frac{2}{\tau[\nu_X + (g^2\gamma+1)\nu]}} - \left(\frac{u_{max}}{\sqrt{K}}\right)^3 \frac{2^{\frac{2}{3}}\tau(\nu_X - (g^3\gamma - 1)\nu)}{3\{\tau[\nu_X + (g^2\gamma+1)\nu]\}^{\frac{3}{2}}}\,. \end{aligned} \tag{9}$$

Hence, up to corrections of order $u_{max}/\sqrt{K}$, which are important only for really small rates (of order $\exp -K$), the implicit equation defining response in the low input/low rate regime is analogous to the equation obtained using the diffusion approximation (Eq. (15) of the main text).

For the intermediate linear region, the argument discussed in the main text (Eq. (16) of the main text) remains valid also in the case of shot-noise inputs. Therefore, for u_{max} of order one, the network response is expected to be linear up to corrections of order $1/\sqrt{K}$.

Combining the above results, we find that, for all the regimes of activity the network encounters as u_{max} varies, the classification of nonlinearities done in the main text remains valid also when the shot-noise structure of the inputs is taken into account.

References

1. Helias M, Deger M, Rotter S, Diesmann M. Instantaneous Non-Linear Processing by Pulse-Coupled Threshold Units. PLOS Computational Biology. 2010;6(9):1–10. doi:10.1371/journal.pcbi.1000929.

2. Richardson MJE, Swarbrick R. Firing-Rate Response of a Neuron Receiving Excitatory and Inhibitory Synaptic Shot Noise. Phys Rev Lett. 2010;105:178102. doi:10.1103/PhysRevLett.105.178102.

3. Chapeton J, Fares T, LaSota D, Stepanyants A. Efficient associative memory storage in cortical circuits of inhibitory and excitatory neurons. Proceedings of the National Academy of Sciences. 2012;109(51):E3614–E3622. doi:10.1073/pnas.1211467109.
