# Supplementary material for: Response nonlinearities in networks of spiking neurons
Source: PLoS Comput Biol. 2020 Sep 17;16(9):e1008165. doi: 10.1371/journal.pcbi.1008165 (PMC7524009; doi:10.1371/journal.pcbi.1008165)
Supplement: S3 Text — (PDF) [file pcbi.1008165.s003.pdf]

Response nonlinearities in networks of spiking neurons

Alessandro Sanzeni[1,2], Mark H. Histed[1], Nicolas Brunel[2,3,*]

1 National institute of Mental Health Intramural Program, NIH, Bethesda, MD, USA
2 Department of Neurobiology, Duke University, Durham, NC, USA
3 Department of Physics, Duke University, Durham, NC, USA

* nicolas.brunel@duke.edu

Supporting information

S3 Text: Constraints on the amplitude of response-onset nonlinearities

In all the examples of response-onset nonlinearities we have shown in the main text, deviations from linear response unravel over a limited range of network rates. This observation contrasts with what is observed in the SSN [1], where nonlinearities emerge at response onset and develop over tens/hundreds of spk/s. In this section, we explore in more detail the differences between the SSN and networks of spiking neurons in this regime, focusing on the monostable scenario in which either the s1 or the s2 solution is the only solution of network activity. We show that neuronal refractoriness gives strong constraints on parameters for which only a single solution exists, and that such constraints, together with the shape of the f-μ curve at low input, strongly constrain the range of firing rates for which onset nonlinearities are observed.

Constraints due to neuronal refractoriness

In spiking networks, neuronal refractoriness can generate the emergence of multiple solutions; requiring a unique response creates constraints which are not present in models, such as the SSN, that do not a include refractory period.

An example of the effect of refractoriness on response-onset nonlinearities is shown in Fig. 8 second row, where connectivity creating only supersaturating (s2) solution in the SSN, also produce s3 and s4 solutions in spiking networks. When s2 solutions are present, they are the only solution in the coupling limit when $g_E > 1/\gamma$, else they coexist with s3 and s4 solutions. In supersaturating solutions, the net effect of recurrent interactions is to suppress excitatory activity. This suppression is mediated in the model by the parameter g_E, which controls the ratio between recurrent excitatory and recurrent inhibitory inputs in excitatory cells. Increasing g_E, while fixing all other parameters, produces stronger suppression of excitatory activity and results in a lower peak of excitatory rate in supersaturating solutions. This effect is seen numerically in spiking networks and analytically in the SSN (see Eq 5.14 in [1]).

Therefore, requiring a unique solution generates constraints in the parameter space which are not present in models that do not a include refractory period and limits the amplitude of response-onset nonlinearities in supersaturating solutions.

Constraints due to the shape of the f-μ curve at low input

As shown in the main text, decreasing coupling strength generates larger response-onset nonlinearities; here we show that, in spiking networks, the shape of the $f-\mu$ curve generates constraints on this increase that are not present in the SSN. We perform our analysis using the Ricciardi model, which can be more easily compared with the SSN, since they both have a fixed $f-\mu$ curve. similar results were found in the LIF network.

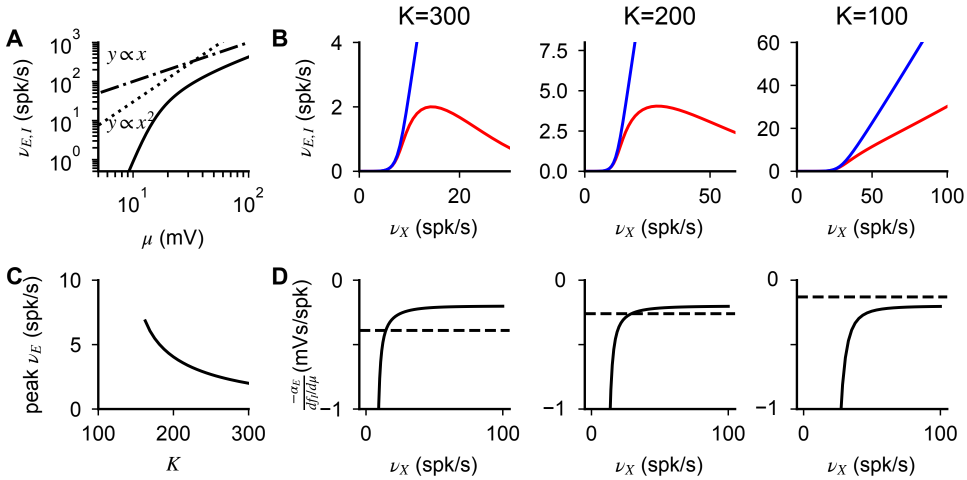

Fig B. Disappearance of supersaturation as the coupling strength decreases (**A**) $f-\mu$ curve of Ricciardi model (black line); it starts supralinearly and becomes linear for large currents. Linear (dash-dotted line) and quadratic (SSN, dotted line) scalings are shown as references. (**B**) Response of excitatory (red) and inhibitory (blue) neurons in Ricciardi model for three different coupling strength (values of $K_{EE} = K_{IE} = K$ indicated on top of the plots). (**C**) Peak response of excitatory neurons in supersaturating solutions as a function of K. Supersaturation disappears for $K \lesssim 160$. (**D**) Values of $-\alpha_E/(df_I/d\mu)$ (continuous lines) and Ω_E/W_{EX} (dashed lines) as a function of ν_X and K. As predicted by Eq. (4), supersaturation emerges only for $\Omega_E/W_{EX} < -\alpha_E/(df_I/d\mu)$; decreasing coupling strength increases the value of Ω_E and prevents supersaturation. Simulation parameters: g_E=4.0;g_I=2.7; $J_{EE} = J_{IE} = J = 0.2$mV; $\alpha_E = \alpha_I = 1$, $W_{EX} = W_{IX} = JK\tau$, $\tau = 20$ms. To simplify comparison with SSN, we assumed $\tau_{rp} = 0$.

Both in the SSN and in the Ricciardi model, firing rates are obtained solving a stationary condition of the form (see methods for details)

$$\nu_A = f(\mu_A)\,, \quad \mu_A = \sum_B W_{AB}\nu_B + W_{AX}\alpha_A\nu_X\,. \tag{1}$$

In the SSN, f is supralinear (specifically, a power-law with exponent~ 2) for any value of μ leading to non-zero firing rates. In the Ricciardi model, f features an expansive nonlinearity, with an exponent larger than two, at low μ, but then becomes linear as μ increases (Fig. B). This difference can affect strongly how the effective coupling between cells changes with the network activity level. In the SSN model, the effective weight from population B to population A is given by

$$\frac{d\nu_A}{d\nu_B} = nk^{\frac{1}{n}}\left[\nu_A\right]^{\frac{n-1}{n}} W_{AB}\,. \tag{2}$$

Therefore, as ν_X increases, no matter how small the Ws are, there is always a critical value at which the effective coupling becomes strong enough that recurrent inputs are

comparable to feedforward inputs. For ν_X below the critical value, the network response is supralinear, as it is determined solely by the single neuron $f-\mu$ curve; for ν_X above the critical value, depending on the structure of the connectivity, the network response can increase linearly or show supersaturation. Decreasing the Ws increases the range of ν_X over which effective coupling is weak and the amplitude of the supralinear response. In the Ricciardi model, applying Eq. (2) locally, around a given value of μ, shows that the effective coupling strength increases with activity only up to the linear region of the $f-\mu$ curves, and saturates in that linear region [1]. Therefore, only a limited decrease in the Ws can augment the range of supralinear response; larger increases produce linear response. This argument shows that, both in regular and supersaturating solutions, the Ricciardi model has an intrinsic limit on how large the region of supralinear response can be. While the supralinear response at low inputs is the only nonlinearity in regular solutions (we are neglecting refractoriness in this section), supersaturating solutions also feature a region of sublinear response which emerges around the point at which recurrent inputs are comparable to feedforward inputs. In what follows, we show that, in the Ricciardi model, also the amplitude of this nonlinearity (specifically, the maximum excitatory rate that can be generated while preserving supersaturation) is limited by the shape of the $f-\mu$ curve. As shown in [1], conditions for supersaturation can be obtained by taking the derivative with respect to ν_X in Eq. (1) and solving for $d\nu_A/d\nu_X$; this gives

$$\frac{d\nu_E}{d\nu_X} = \frac{(W_{EX}\alpha_E + \Omega_E \; df_I/d\mu) \;\; df_E/d\mu}{\det \mathcal{W}} \,, \quad \Omega_E = W_{II}W_{EX}\alpha_E - W_{EI}W_{IX}\alpha_I \,, \qquad (3)$$
$$\mathcal{W}_{AB} = \delta_{AB} - W_{AB}\; df_A/d\mu \,, \quad df_A/d\mu = df/d\mu\big|_{\mu_A} \,.$$

The excitatory rate ν_E decreases with ν_X, i.e. supersaturation emerges, any time that the r.h.s. of Eq. (3) is negative. In the SSN, dynamical stability of the model requires $\det\mathcal{W} > 0$ [1]. In the Ricciardi model, $W_{AB} = \tau_A K_{AB} J_{AB}$, and

$$\det \mathcal{W} = 1 + df_I/d\mu \; df_E/d\mu \tau_E \tau_I K^2 J^2 \beta\gamma (g_E - g_I) + KJ\left(\tau_I \beta\gamma g_I df_I/d\mu - \tau_E df_E/d\mu\right) \,.$$

Since, in supersaturating solutions, $g_E > g_I$ (Eq. (29) of the main text) and $df_I/d\mu > df_E/d\mu$, $\det\mathcal{W}$ is expected to be greater than zero for a broad parameter range also in the Ricciardi model. Therefore, both in the SSN and in the Ricciardi model, supersaturation emerges if

$$\Omega_E < -\frac{W_{EX}\alpha_E}{df_I/d\mu} \,. \qquad (4)$$

In regular and supersaturating solutions, the inhibitory rate ν_I and μ_I increase monotonically with ν_X. In the SSN, because of the supralinearity of the $f-\mu$ curve, $df_I/d\mu$ increases with ν_X and, if $\Omega_E < 0$, there is always a value of ν_X at which Eq. (4) is satisfied, no matter how small the Ws are. In the Ricciardi model, $df_I/d\mu$ increases with μ_I only up to the linear region of the $f-\mu$ curve and Eq. (4) can be satisfied only if the Ws are large enough to give $\Omega_E < -W_{EX}\alpha_E/\max\{df_I/d\mu\}$. Solving numerically Eq. (1) in the Ricciardi model (Fig. B), we find, in agreement with the above argument, that ν_E decreases with ν_X only when Eq. (4) is satisfied. Furthermore, a moderate reduction in coupling strength increases the value of ν_X at which supersaturation emerges and increases the peak excitatory rate. Supersaturation eventually disappears as coupling strength decreases.

[1] Note that in this figure we use $\tau_{rp} = 0$. In the presence of a refractory period $\tau_{rp} > 0$, the effective coupling strength reaches a maximum and then gradually decreases as the neuron gets closer to saturation.

To summarize, in the Ricciardi model, the range of rates over which response-onset nonlinearities unravel increases for a moderate decrease in coupling strength, and linear response emerges for more significant reductions. This property limits the amplitude of nonlinearities generated at response-onset.

References

1. Ahmadian Y, Rubin DB, Miller KD. Analysis of the stabilized supralinear network. Neural Computation. 2013;25(8):1994–2037. doi:10.1162/NECO.
